# Detection of human cytomegalovirus in glioblastoma among Taiwanese subjects

**DOI:** 10.1371/journal.pone.0179366

**Published:** 2017-06-08

**Authors:** Ching-Fen Yang, Hsiang-Ling Ho, Shih-Chieh Lin, Chih-Yi Hsu, Donald Ming-Tak Ho

**Affiliations:** 1 Department of Pathology and Laboratory Medicine, Taipei Veterans General Hospital, Taipei, Taiwan; 2 School of Medicine, National Yang-Ming University, Taipei, Taiwan; University of St Andrews, UNITED KINGDOM

## Abstract

The relationship between human cytomegalovirus (HCMV) and glioblastoma (GBM) has been debated for more than a decade. We investigated the presence of HCMV genes, RNA and protein in GBMs and their relationships with tumor progression. Results of quantitative PCR for HCMV UL73, nested PCR for HCMV UL144, in situ hybridization (ISH) for RNA transcript, and immunohistochemistry (IHC) for protein expression and their relationship to the prognosis of 116 patients with GBM were evaluated. Nine (7.8%) cases revealed a low concentration of HCMV UL73, and only 2 of the 9 (1.7%) cases showed consistent positivity on repeat PCR testing. HCMV UL144, ISH and IHC assays were all negative. The HCMV UL73 positive cases did not show significant difference in the clinicopathological characters including age, gender, Karnofsky performance status, extent of resection, bevacizumab treatment, isocitrate dehydrogenase 1 mutation, O^6^-methylguanine-DNA-methyltranferase status and Ki67 labeling index, and did not reveal prognostic significance. As only one HCMV gene was detected at low concentration in 7.8% of GBMs and there was no evidence of transcription, protein expression or prognostic impact, we cannot conclude a relationship between HCMV and GBM in Taiwanese patients.

## Introduction

Glioblastoma (GBM) is the most common malignant neoplasm of the central nervous system. Patients’ survival has significantly improved since the use of radiotherapy with concomitant and adjuvant temozolomide (TMZ) chemotherapy and its median survival is 20.0 months at our institute [1, 2]. However, in comparing with other common malignancies in Taiwan, such as colon and breast cancers, its survival is still poor. Therefore, studies to identify new therapeutic targets and/or better managements for GBM are needed.

The seroprevalence of human cytomegalovirus (HCMV) is high [3] and the virus establishes a lifelong latency in the host with periodic reactivations [4]. It is a neurotropic virus, which can infect brain microvascular endothelial cells, astrocytes, pericytes, neurons, microglial cells, neural stem cells and neural precursor cells, and impact neuronal differentiation in fetuses [4–6]. HCMV can also cross brain endothelium by virus-infected leukocytes [7]. In 2002, Cobbs, et al. have demonstrated that HCMV was commonly detected in GBM [8], and suggested that HCMV gene products could promote GBM pathogenesis. The HCMV US28 protein is a chemokine receptor that promotes angiogenesis and tumor formation via activation of NFkB and inducing cyclooxygenase-2 (COX-2) expression [9]. Colocalization of US28 and phosphorylated STAT3 (p-STAT3) in GBM and poor prognosis of p-STAT3 abundant GBM had also been reported [10]. However, the reported prevalence of HCMV in GBMs ranged from 0% to 100% [11–27]. The roles of HCMV in glioma development and progression are also controversial in recent years [28]. A study has demonstrated that GBM patients with low-grade HCMV infection were associated with longer survival [29]. A randomized trial, which included 42 GBM patients and 22 of them received anti-viral therapy (valganciclovir), showed no survival benefit in the study period of 6 months [30]. However, prolonged overall survival in patients receiving more than 6 months of valganciclovir was reported [30]. Therefore, the relationship between HCMV and GBM should be seriously evaluated. Moreover, Cobbs, et al. has also provided detailed methodology for detection of HCMV in GBMs that they used in order to sort out the controversy in the literature [31].

In this study, we intended to use the methods that Cobbs, et al. described to study our GBMs. Protein expression by immunohistochemical stains and RNA transcript by in situ hybridization were used to confirm the viral DNA copies detected by PCR, and their relationships with tumor progression were evaluated.

## Materials and methods

### Patients

The study protocol was approved by the Institutional Review Board (IRB) of the Taipei Veterans General Hospital, Taiwan, ROC, and the IRB waived the need for written informed consent. One hundred and sixteen patients with primary GBM were retrieved from the surgical pathology file collected from October, 2007 to January, 2014. These patients had received post-operative radiotherapy with concomitant and six cycles of adjuvant TMZ chemotherapy and had adequate follow-up data. The general data of the patients, including age, gender, Karnofsky performance status (KPS), date of surgery, extent of resection, history of radiotherapy and medication were reviewed from the medical records. A 25% or more increase in size of enhancing tumor or any new tumor on magnetic resonance imaging (MRI) was considered as progression according the Macdonald criteria [32]. For each patient, the original histopathology slides were reviewed for confirmation of the diagnosis. The data and samples were analyzed anonymously after a coding procedure.

### Quantitative real-time PCR (qPCR) of HCMV UL73 gene

Genomic DNA was isolated from paraffin-embedded tissue using the PicoPure DNA extraction kit (Applied Biosystems, Foster City, CA, USA). The BIOMED-2 protocol was used to screen the quality and amplifiability of the isolated DNA.

UL73 plasmid DNA control was generated as previously described [33]. Sequences of primers and the TaqMan fluorogenic probe used for qPCR were as follows: forward primer UL73-C-F 5’TGGTGGACTATGCTTAACGCTC3’, reverse primer UL73-C-R 5’TCTGGAAGCAGCAATGTCGTA3’, and TaqMan MGB probe 5’ATTCTGATGGGAGCTTTT3’ carrying a 5’ FAM reporter dye and a 3’ quencher dye. In brief, 2 μl of genomic DNA or UL73 plasmid DNA control, at a concentration of 100 ng/μl, was added to a 20μl of real-time PCR reaction containing 2 X TaqMan PCR Universal Master Mix, 300 nM each primer, 200 pmole fluorogenic probe. Each qPCR reaction was performed in duplicate. The qPCR condition was set as 50°C for 2 min and 95°C for 10 min, followed by 45 cycles of 95°C for 15 s and 60°C for 1 min. The number of UL73 copies was deduced from the PCR threshold cycle (Ct), which was defined as the fractional cycle number at which the fluorescence reaches 10 times the standard deviation (SD) of the baseline, and was determined by the qPCR software. Samples with an average Ct value ≤ 40.2 cycles (≥ 1.0 copies/200 ng) were considered as positive for the presence of HCMV. A previously immunohistochemistry (IHC) proven HCMV gastritis was used as positive control, and a normal brain was used as negative control. The assay was validated and matched with the results of HCMV IHC in 6 HCMV+ and 7 HCMV–formalin-fixed and paraffin-embedded (FFPE) gastric and cerebral specimens. Of note, the reason for detecting HCMV UL73 gene rather than US28 gene, which was used in the study of Soroceanu et al. [31, 34] was the higher sensitivity of detecting UL73 gene (100%, 6/6 HCMV IHC positive samples) than US28 gene (83.3%, 5/6) in our preliminary study (S1 Table).

### Nested PCR Amplification of HCMV UL144 gene

Two μl (200 ng) of genomic DNA were subjected to first-round PCR amplification with the external forward primer 5’GGCATCTCTCACTCCGATAGG3’ and reverse primer 5’GTGCGCACCTAAGAACCATACG3’. One μl of amplified PCR products were then used as templates for second-round PCR amplification with nested forward primer 5’GAGAGACAACCAGGCTAGAG3’ and reverse primer 5’CAACATCACAAGCAACGACAGC3’. Four μl of the nested PCR products were mixed with fluorescent dye, subsequently separated with 2% agarose gel electrophoresis, and visualized under UV illumination. A previously IHC proven HCMV gastritis was used as positive control, and a normal brain was used as negative control. The assay was validated and completely matched with the results of HCMV IHC in 6 HCMV+ and 7 HCMV–formalin-fixed and paraffin-embedded (FFPE) gastric and cerebral specimens.

### In situ hybridization (ISH)

ISH was performed using in vitro diagnostic HCMV fluorescein-conjugated oligonucleotide probe (Bond^™^ Ready-to-Use ISH CMV Probe, Catalog No: PB0614, Leica biosystem, Newcastle, UK) to identify early gene RNA transcript according to the manufacturer’s recommendation on BOND-MAX immunostainer (Leica Microsystems). The procedures were as follows: (1) deparaffinization of tissue on the slides with Bond Dewax Solution (Leica Microsystems) at 72°C for 30 minutes, (2) tissue pretreatment with Bond Enzyme 1 (Leica Microsystems) for 15 minutes at 37°C, (3) incubation with HCMV probe over night at 37°C, (4) incubation with Anti-Fluorescein Antibody (Leica Microsystems) for 20 minutes at ambient temperature, (5) incubation with Post Primary reagent (Leica Microsystems) for 8 minutes at ambient temperature, followed by washing with Bond Wash solution (Leica Microsystems) for 6 minutes, (6) Bond Polymer (Leica Microsystems) for 8 minutes at ambient temperature, followed by washing with Bond Wash and distilled water for 4 minutes, (7) peroxidase for 5 minutes, (8) color development with 3,3’-diaminobenzidine tetrahydrochloride for 5 minutes at ambient temperature, and (9) hematoxylin counterstaining for 5 minutes at ambient temperature, followed by mounting of the slides. A previously proven cytomegalovirus gastritis was used as positive control, and intense, brown predominately cytoplasmic staining was regarded as positive (S1 Fig). All slides were read by CFY, DMH and CYH.

### Immunohistochemical study (IHC)

Tissue sections were immunostained using anti-HCMV IE1/IE2 antibody, clone 8B1.2 (1:40; Millipore, Temecula, CA) and followed the protocol of Cobbs et al. [31]. In brief, deparaffinized tissue sections (6 μm thick) were performed post fixation with 10% normal buffered formalin, pepsin digestion, heat antigen retrieval and blocking with endogenous peroxidase, avidin, biotin, Fc receptor, and then the sections were incubated with primary antibody in a humidified container overnight at 4–8°C. Visualization was carried out using goat anti-mouse secondary antibody (1:18, BioGenex Laboratories, San Ramon, CA, USA), peroxidase-labeled streptavidin and 3,3'-diaminobenzidine. All sections were counterstained with hematoxylin. A previously proven cytomegalovirus gastritis was used as positive control, and nuclear staining was regarded as positive (S2 Fig). All slides were read by CFY, DMH and CYH.

### Statistical analysis

The Fisher’s exact test was used to compare the distribution of categorical variables. Differences in continuous variables were compared by Mann–Whitney Wallis test. Progression-free survival (PFS) was measured from the date of surgery to the date of progression. Overall survival (OS) was measured from the date of surgery to the date of death or last follow-up. PFS and OS curves were plotted by Kaplan-Meier method, and their differences were calculated by log-rank test. Cox regression model was used to adjust the influence of age, gender, KPS, extent of resection, bevacizumab treatment, isocitrate dehydrogenase 1 (IDH1) mutation and O^6^-methylguanine-DNA-methyltranferase (MGMT) status. The data of the MGMT status were from our previous study [2] and samples with methylation-specific PCR products of any intensity were regarded as a positive result. *P*-values were derived from two-tailed tests and *p* < 0.05 was considered significant.

## Results

The clinical characteristics of the patients are listed in the S2 Table. Nine of 116 (7.8%) cases showed positivity for HCMV UL73 qPCR, and the Ct values ranged from 35 to 40 cycles (Table 1). For each genomic DNA fraction of 200 ng, the copy number of HCMV UL73 gene ranged from 1.2 to 38.2 (median 3.5) copies. However, the ISH and IHC of these 9 HCMV UL73 positive cases were negative.

**Table 1:** Characteristics of the glioblastomas with HCMV_UL73 positivity.

Age (yr)	Sex	UL73 (Ct)	UL73 repeat (Ct)	UL144	IHC	ISH	MSP	IDH1	Ki67 (%)	PFS (mo)	OS (mo)
67	m	35	36	–	–	–	+	–	25	10.3	16.7
40	m	39	40	–	–	–	+	R132H	85	2.9	13.3
9	f	38	–	–	–	–	–	–	70	5.6	14.6
72	m	38	–	–	–	–	–	–	50	5.5	13.1
56	f	38.3	–	–	–	–	+	–	40	8.4	15.5
75	m	38.4	–	–	–	–	+	–	90	16.4	20.3
37	f	39	–	–	–	–	+	–	20	7.6	23.9
54	f	39	–	–	–	–	–	–	70	4.7	10.7
57	m	40	–	–	–	–	–	–	60	3.1	8.9

Ct, PCR threshold cycle; MSP, MGMT methylation specific PCR; IDH1, isocitrate dehydrogenase 1 mutation; PFS, progression-free survival; OS, overall survival.

The clinicopathological characters, including age, gender, KPS, extent of resection, bevacizumab treatment, IDH1 status and MGMT status, did not show significant difference between the HCMV UL73 positive and negative cases (Table 2). The median of Ki67 labeling index of the HCMV UL73 positive cases (60%) was higher than that of the HCMV UL73 cases (45%) (S3 Fig), but the difference did not reach statistical significance (*p* = 0.327).

**Table 2:** Comparisons of HCMV_UL73 positive and negative cases.

	Total	UL73+	UL73–	*p*
N	116 (100%)	9 (100%)	107 (100%)	
Age (year)	55	56	55	0.857
Male	69 (59%)	5 (56%)	64 (60%)	1.000
KPS≥80	63 (54%)	5 (56%)	58 (54%)	1.000
Total resection	94 (81%)	7 (78%)	87 (81%)	0.679
Bevacizumab+	25 (22%)	2 (22%)	23 (21%)	1.000
IDH1+	10 (9%)	1 (11%)	9 (8%)	0.569
MSP+	59 (51%)	5 (56%)	54 (50%)	1.000
Ki67 (%)	45	60	45	0.327
PFS (month)	7.5	5.6	7.5	0.147
OS (month)	18.0	14.6	19.2	0.141

Data presented as N (%) or median

KPS, Karnofsky performance status; IDH1, isocitrate dehydrogenase 1; MSP, MGMT methylation specific PCR; PFS, progression-free survival; OS, overall survival.

**Fig 1:**
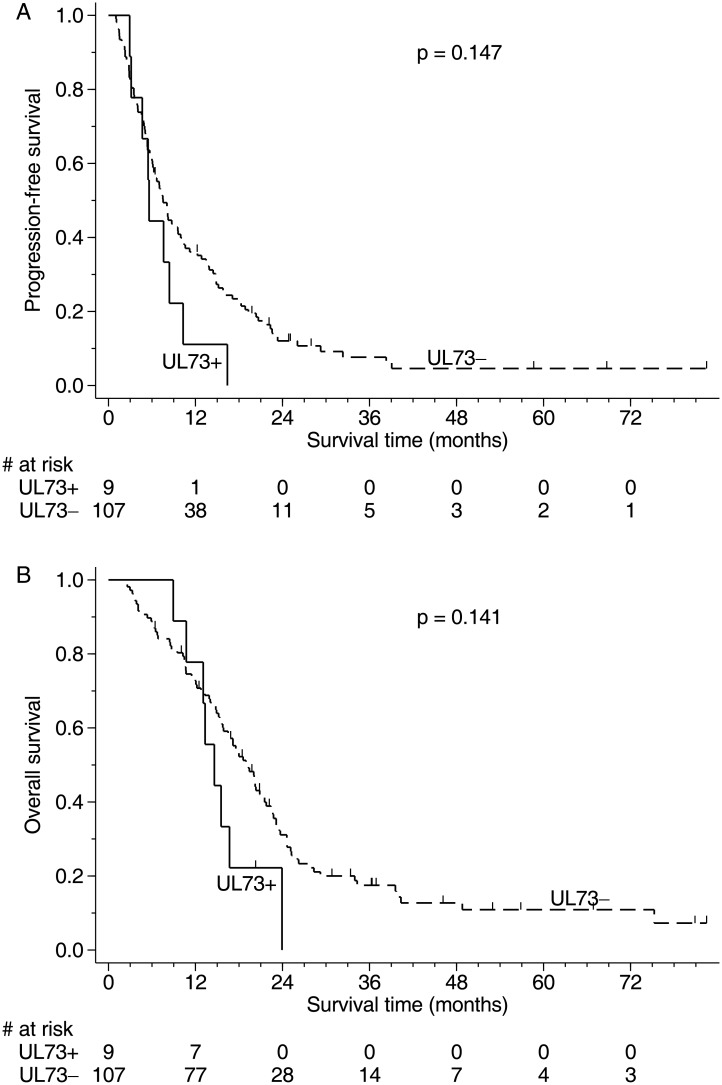
Survival curves of GBM patients stratified by the results of HCMV UL73. (A) The median progression-free survival of HCMV UL73 positive cases (5.6 months) was shorter than that of HCMV UL73 negative cases (7.5 months). However, their difference was not significant (*p* = 0.147). (B) The median overall survival of HCMV UL73 positive cases (14.6 months) was shorter than those of HCMV UL73 negative cases (19.2 months). However, their difference was not significant (*p* = 0.141).

Although the medians of PFS and OS of the HCMV UL73 positive cases were shorter than those of the HCMV UL73 negative cases, their differences were not significant (Fig 1). Cox regression model with adjustment of the influence of age, gender, KPS, extent of resection, bevacizumab treatment, IDH1 status and MGMT status, HCMV UL73 positivity did not reveal the prognostic significance in both PFS and OS (*p* = 0.122 and *p* = 0.193).

Due to low copy number of the HCMV UL73 gene in the nine cases with positive qPCR result, we repeated the HCMV UL73 test for further confirmation. Only two of the nine cases got positive result in repeat HCMV UL73 tests and their HCMV copy numbers were 1.2 and 19.1 copies / 200 ng. Nested PCR assay for HCMV UL144 of all the 116 cases was negative.

## Discussion

HCMV is a DNA virus of the family Herpesviridae. After primary infection of HCMV, the virus persists in latency and involves expression of specific latency-associated viral gene products in the latently infected cell [35]. Since Cobbs and colleagues reported the presence of HCMV in malignant gliomas [8], the relationship between HCMV and GBM has been debated for more than a decade. Several studies reported high prevalence of HCMV in GBMs [11–19], while the others failed to identify any HCMV [20–27]. The discrepancy could be the differences in the sensitivities of the assays employed in different studies.

In this study, we evaluated HCMV in 116 GBM by qPCR for the viral gene UL73, nested PCR for UL144, ISH and IHC, and only 9 (7.8%) cases revealed a low concentration of viral DNA copies by qPCR. The reason to choose HCMV UL73 and UL144 in the present study was based on their sensitivity of the primers. The qPCR for HCMV UL73 gene was very sensitive and it could detect ≥ 1.0 copies/200 ng sample. Its sensitivity was better than that for detecting HCMV US28 gene (S1 Table). Our colleagues from our department had detected HCMV UL73 gene in 42.2% (35/83) of the colorectal tumor samples [36] and shown good correlation with the result of PCR for HCMV UL55 in 23 pairs of colorectal cancer and adjacent non-neoplastic specimen [33]. In regard to the age of the specimen, Ranganathan et al. had demonstrated that detecting HCMV UL144 gene in GBMs was more sensitive than detecting UL28 and UL55, especially for older formalin-fixed, paraffin-embedded (FFPE) samples [37]. The HCMV UL73 gene assay had also been validated and did not show positivity in non-neoplastic cerebral controls [33]. In our study, only 2 of the 9 (22.2%) UL73 gene positive cases showed a consistent positivity in the repeat qPCR tests, suggesting that the viral gene within these tumors was few and it was not correlated with viral protein expression by IHC. Although nested PCR is very sensitive and could be served as a screening tool, our nested PCR assays for UL144 were all negative. This could be attributed to variations at primer/probe binding sites and low viral DNA copy numbers. The primers that we used for UL144 were the same as our colleagues used to study colorectal cancer, of which they detected UL144 gene in 42.6% (49/230) of colorectal carcinomas and in 11.3% (13/230) of adjacent non-neoplastic specimens [38].

Due to the negative results of this investigation, the potential etiology of false negative results including the measurement errors and small sample size might be concerning. In this study, the immunohistochemical stains were strictly followed the methodology by Cobbs, et al., which included section thickness, deparaffinization method, post-fixation, pepsin digestion, antigen retrieval method, blocking methods, antibody concentration and incubation. Proper staining results were observed in both positive and negative control tissues. However, all the studied GBMs showed negative staining. As the effectiveness of ISH biotinylated probes could be affected by tissue with high endogenous biotin expression, we used an in vitro diagnostic HCMV fluorescein-conjugated probe and performed on autostainer. Negative results were seen in all the study cases, which were accompanied with proper positive control. In addition to the probes, denaturation, hybridization and detection could be different, and might account for the different outcomes. Similar to our results, a recent report showed no expression of HCMV in 32 GBM by IHC and RT-PCR HCMV assay [39].

Although tissue fixation/processing might mask antigen and have influence on IHC staining results, a previous study did not reveal significant differences between FFPE tissue and frozen tissue. Of the nine GBMs that they studied, one case showed positivity in both FFPE and frozen tissues, one showed positivity on FFPE tissue, while it was negative in frozen tissue, and the rest were negative in either FFPE or frozen tissues. (Fisher's exact test *p* = 0.22) [11]. They also suggested that IHC with anti-IE1 antibody was the most reliable and the simplest technique for detecting HCMV on fixed or frozen tissue sections [11]. However, Yamashita et al using liquid chromatography-tandem mass spectrometry analysis revealed that proteins extracted from the IE1 and pp28 positive bands were non-viral human proteins such as human serum albumin (HSA) and myelin basic protein (MBP), suggesting previously unknown cross-reactivity of these antibodies [24]. Furthermore, a large-scale transcriptome sequencing study of 167 GBMs from the Cancer Genome Atlas Research Network found absence of relevant HCMV expression [22]. RNA-Seq by next-generation sequencer revealed no viral RNA from DNA viruses such as HCMV was present in glial tumors [40]. To our knowledge, there has been no HCMV viral particle ever isolated from glioma tissue to date.

From the previous reports, the relationship between HCMV and GBM did not show significant differences among populations with different ethnic origins. Of the studies from the United States, although several reports supported their relationship [8, 12–14, 41, 42], others did not [20, 23, 43]. The reports from Sweden were also conflicting [19, 22, 27, 29, 44]. In the Far East region, reports from China showed high prevalence of HCMV viral protein in GBMs [17, 18], while reports from Japan were unable to demonstrate the presence of HCMV genome in GBMs [24, 26].

There has been no consistent correlation between HCMV seroprevalence rates and GBM incidence rates. An epidemiology study in the United States showed HCMV seroprevalence is significantly lower in whites than in blacks or Hispanics, while the incidence of GBM is higher in whites [45]. The HCMV seroprevalence in the United States is 45.2%– 55.5%, which is much lower than that in Taiwan being 91.1% [46], however, the annual incidence of malignant glioma in the United States is more than double of that in Taiwan (5 cases per 100,000 population vs. 2 cases per 100,000 population) [47]. Therefore, the correlation HCMV seroprevalence and GBM cannot be substantiated.

It is possible that detection of HCMV in GBM tissues is related to latent HCMV reactivation secondary to treatment-related immunosuppression. Of the previous works that detected HCMV in tumors and had detailed treatment information, none of them had received radiotherapy or chemotherapy prior to surgery [13, 15, 17, 18]. Our patients also did not receive any radiotherapy or chemotherapy before removal of the tumors. Therefore, viral reactivation secondary to treatment-related immunosuppression is not likely.

Despite HCMV could be a pathogen or bystander for gliomagenesis, some studies also proposed that HCMV might be oncomodulatory and enhance tumor progression by a specific mechanism [48, 49]. CMV proteins could control cell cycle, induce telomerase activity, inhibit apoptosis, induce angiogenesis, activate cell migration and metastasis, avoid immune destruction, increase genome instability, and promote stemness by blocking cellular differentiation [50, 51]. As the consequence, presence of CMV in GBMs would promote tumor progression and associate with a worse prognosis [29, 42, 52]. However, our results revealed a low concentration of HCMV UL73 gene in 7.8% of GBMs and had no prognostic impact on PFS and OS. The later could be a statistical bias caused by a small sample size of HCMV UL73 positive cases. Further studies including more HCMV UL73 positive cases are needed to clarify this issue. However, there was also no prognostic difference reported from the data of Chinese Glioma Genome Atlas [17]. The Swedish trial also did not show clear clinical benefit of anti-viral therapy for GBM and survival benefit [30]. All these results showed insufficient evidence to recommend routine testing for CMV in GBM or to treat HCMV as an add-on therapy.

Besides the usual limitations of a retrospective study, the following restrictions should be considered when interpreting the results of this study. First, as HCMV+ GBM was not available, HCMV gastritis was used as the IHC positive control. This may not be ideal as the viral load in these two conditions and the method of fixation/tissue preparation of these two types of tissues could be different. Second, since we only studied HCMV UL73 and UL144, the possibility of existence of HCMV could not be totally excluded as different primer/probe binding sites could exist. Third, the case number of HCMV UL73 positive cases was small and the prognostic impact of which could not be confidently assessed.

In conclusion, our results revealed a very low concentration of HCMV UL73 gene present in 7.8% of our 116 GBMs studied. However, HCMV RNA and protein were not detected. The results suggested that HCMV is unlikely to be implicated in the development of GBMs at least in the Taiwanese cases.

## Supporting information

S1 Fig Positive control of in situ hybridization. A case of cytomegalovirus gastritis was used as positive control for in situ hybridization. Intense, brown predominately cytoplasmic staining was regarded as positive.
(TIF)

S2 Fig Positive control of immunohistochemical (IHC) stain. A case of cytomegalovirus gastritis was used as positive control for IHC stain. Nuclear staining was regarded as positive.
(TIF)

S3 Fig Examples of Ki67 immunohistochemical stain. (A) An HCMV UL73 positive case (Ki67 labeling index = 78%), and (B) an HCMV UL73 negative case (Ki67 labeling index = 45%).
(TIF)

S1 Table Results of detecting UL73 gene and US28 gene in gastrointestinal tract and brain tissues. (PDF)

S2 Table Clinico-pathological features of the analyzed patients. (PDF)
