# Supplementary figures and images for: Detection of human cytomegalovirus in glioblastoma among Taiwanese subjects
Source: PLoS One. 2017 Jun 8;12(6):e0179366. doi: 10.1371/journal.pone.0179366 (PMC5464665; doi:10.1371/journal.pone.0179366)

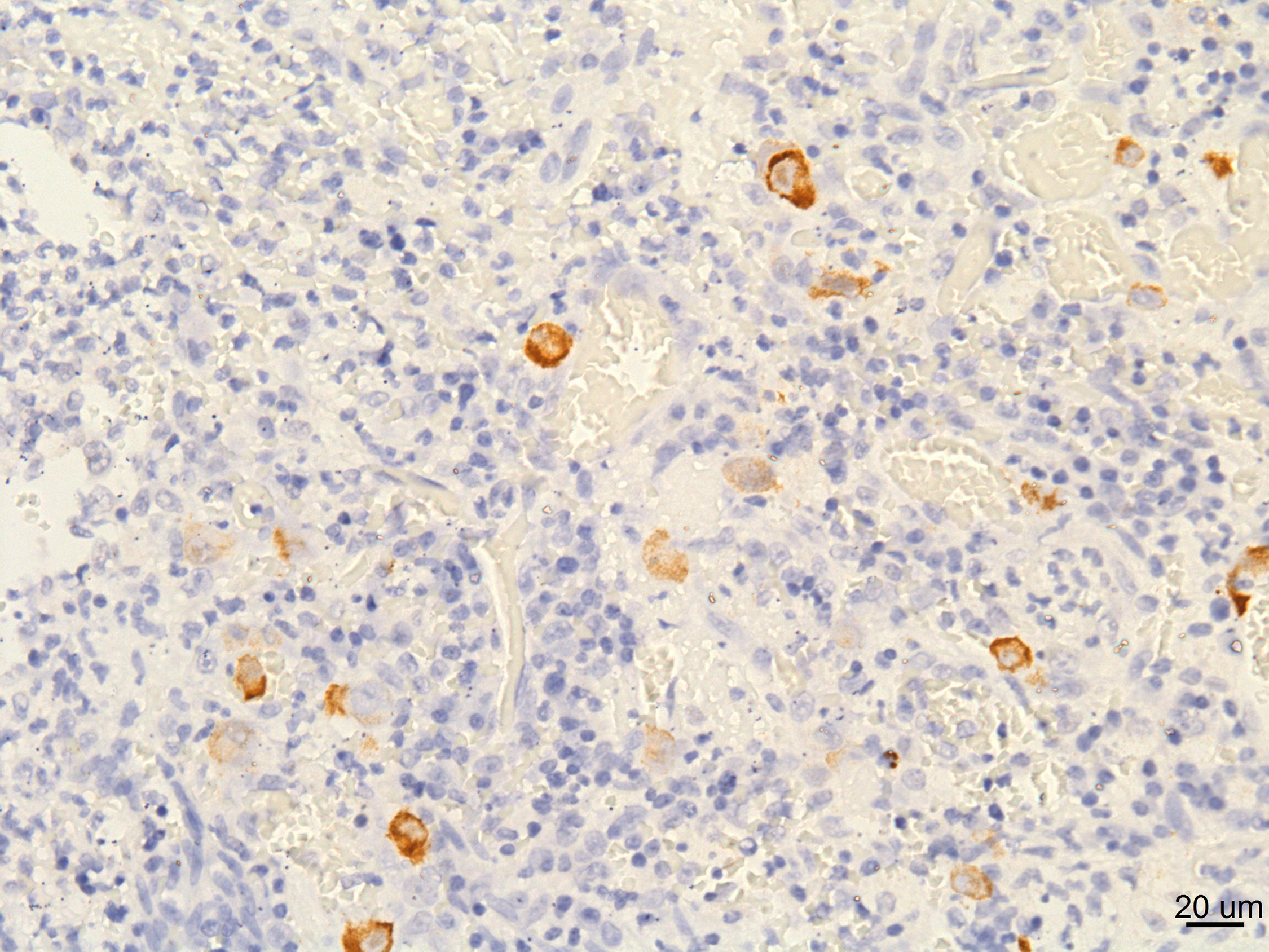

Supplement: S1 Fig — A case of cytomegalovirus gastritis was used as positive control for in situ hybridization. Intense, brown predominately cytoplasmic staining was regarded as positive. (TIF) [file pone.0179366.s001.tif]

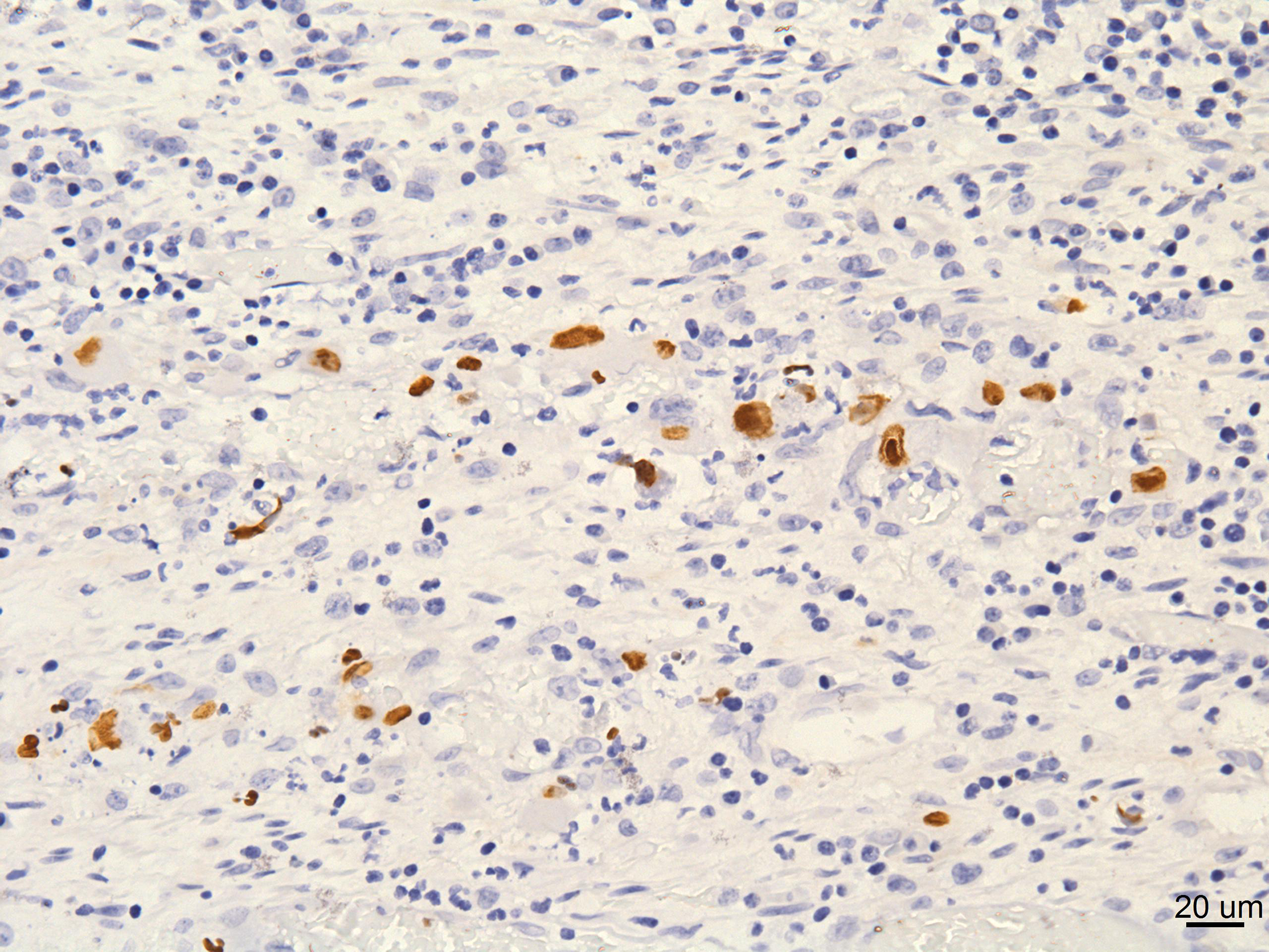

Supplement: S2 Fig — A case of cytomegalovirus gastritis was used as positive control for IHC stain. Nuclear staining was regarded as positive. (TIF) [file pone.0179366.s002.tif]

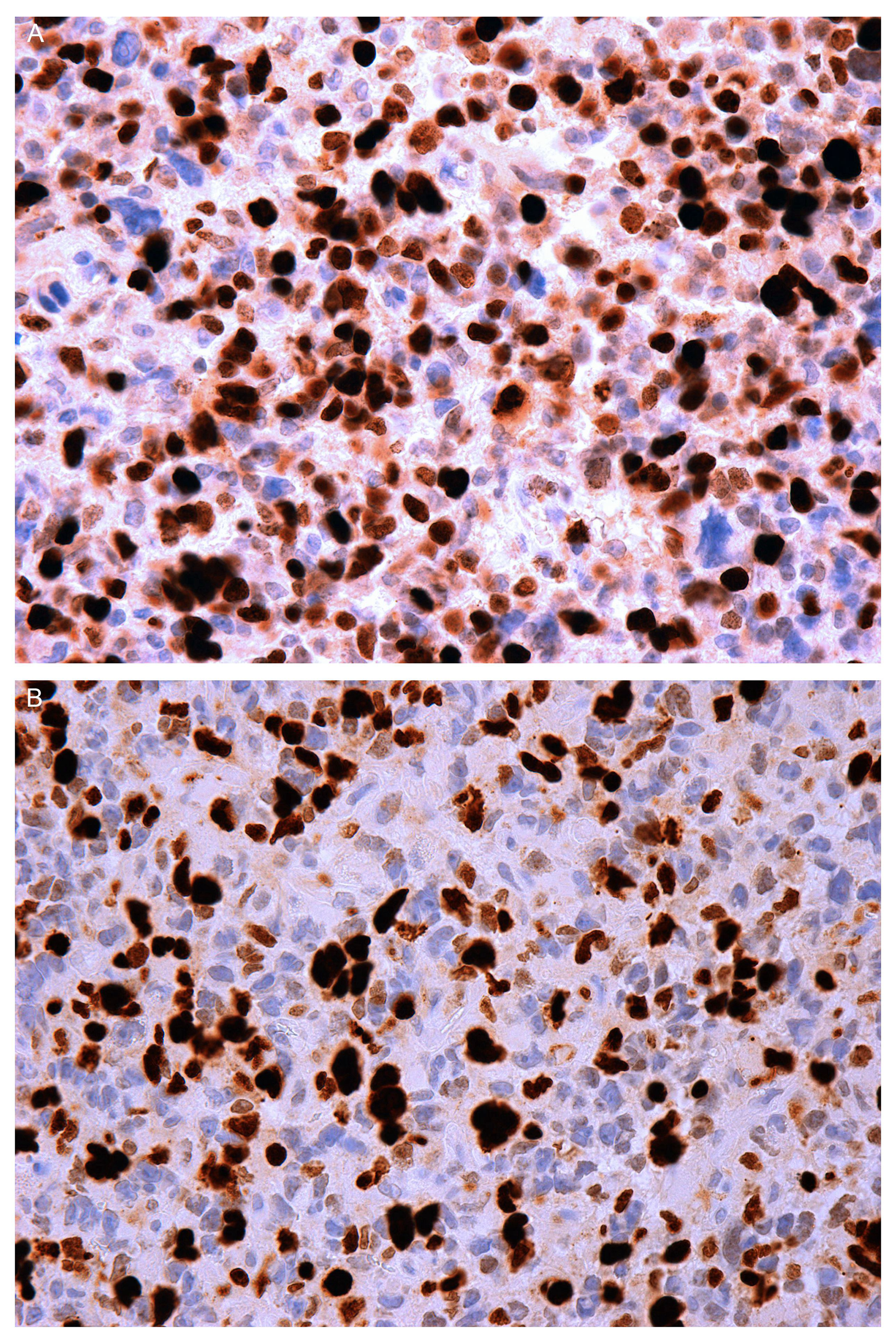

Supplement: S3 Fig — (A) An HCMV UL73 positive case (Ki67 labeling index = 78%), and (B) an HCMV UL73 negative case (Ki67 labeling index = 45%). (TIF) [file pone.0179366.s003.tif]
