# Supplementary material for: Detection of human cytomegalovirus in glioblastoma among Taiwanese subjects
Source: PLoS One. 2017 Jun 8;12(6):e0179366. doi: 10.1371/journal.pone.0179366 (PMC5464665; doi:10.1371/journal.pone.0179366)
Supplement: S1 Table — (PDF) [file pone.0179366.s004.pdf]

S1 Table. Results of detecting UL73 gene and US28 gene in gastrointestinal tract and brain tissues.

Tissue with immunohistochemistry proven HCMV infection			
Tissue	Diagnosis	UL73 Ct value	US28 Ct value
Esophagus	HCMV*	39.25	Undetermined
Stomach	HCMV*	25.26	32.14
Stomach	HCMV*	27.29	24.39
Colon	HCMV*	26.25	39.37
Colon	HCMV*	33.44	35.65
Colon	HCMV*	21.43	25.79
Tissue with no known HCMV infection			
Tissue	Diagnosis	UL73 Ct value	US28 Ct value
Cerebrum	Glioblastoma	36.38	Undetermined
Cerebrum	Glioblastoma	Undetermined	Undetermined
Cerebrum	Glioblastoma	Undetermined	Undetermined
Cerebrum	Glioblastoma	Undetermined	Undetermined
Cerebrum	Hematoma	Undetermined	Undetermined
Cerebrum	Gliosis	Undetermined	Undetermined
Colon	Inflammation	Undetermined	Undetermined
Colon	Inflammation	Undetermined	Undetermined

*HCMV, human cytomegalovirus (immunohistochemical stain positive)
