# Supplementary material for: Detection of human cytomegalovirus in glioblastoma among Taiwanese subjects
Source: PLoS One. 2017 Jun 8;12(6):e0179366. doi: 10.1371/journal.pone.0179366 (PMC5464665; doi:10.1371/journal.pone.0179366)
Supplement: S2 Table — (PDF) [file pone.0179366.s005.pdf]

**S2 Table. Clinico-pathological features of the analyzed patients**

| Age | Sex | UL73 | UL73 repeat | MSP | IDH1 | Ki67 | Total resection | KPS80 | Bevacizumab | PFS | Progress | OS | Death |
|---|---|---|---|---|---|---|---|---|---|---|---|---|---|
| 67 | m | 35 | 36 | 1 | 0 | 25 | 0 | 1 | 0 | 10.3 | 1 | 16.7 | 1 |
| 40 | m | 39 | 40 | 1 | 1 | 85 | 1 | 0 | 0 | 2.9 | 1 | 13.3 | 1 |
| 9 | f | 38 | - | 0 | 0 | 70 | 1 | 1 | 1 | 5.6 | 1 | 14.6 | 1 |
| 72 | m | 38 | - | 0 | 0 | 50 | 1 | 0 | 0 | 5.5 | 1 | 13.1 | 1 |
| 56 | f | 38.3 | - | 1 | 0 | 40 | 1 | 1 | 0 | 8.4 | 1 | 15.5 | 1 |
| 75 | m | 38.4 | - | 1 | 0 | 90 | 1 | 1 | 0 | 16.4 | 1 | 20.3 | 0 |
| 54 | f | 39 | - | 0 | 0 | 70 | 1 | 0 | 0 | 4.7 | 1 | 10.7 | 1 |
| 37 | f | 39 | - | 1 | 0 | 20 | 0 | 1 | 0 | 7.6 | 1 | 23.9 | 1 |
| 57 | m | 40 | - | 0 | 0 | 60 | 1 | 0 | 1 | 3.1 | 1 | 8.9 | 1 |
| 46 | m | - | - | 0 | 0 | 75 | 1 | 1 | 1 | 18.9 | 1 | 46.0 | 0 |
| 39 | f | - |  | 1 | 0 | 30 | 0 | 0 | 0 | 1.3 | 1 | 10.5 | 1 |
| 44 | f | - |  | 0 | 0 | 20 | 0 | 0 | 0 | 1.5 | 1 | 4.1 | 1 |
| 66 | f | - |  | 0 | 0 | 60 | 1 | 1 | 0 | 1.5 | 1 | 8.4 | 1 |
| 15 | f | - |  | 0 | 0 | 80 | 1 | 0 | 1 | 2.5 | 1 | 4.9 | 1 |
| 5 | f | - |  | 0 | 0 | 90 | 0 | 0 | 0 | 2.6 | 1 | 2.6 | 1 |
| 58 | f | - |  | 0 | 0 | 50 | 1 | 0 | 1 | 2.9 | 1 | 3.6 | 1 |
| 7 | f | - |  | 0 | 0 | 80 | 1 | 0 | 1 | 3.0 | 1 | 13.3 | 1 |
| 65 | f | - |  | 1 | 0 | 35 | 0 | 0 | 0 | 3.1 | 1 | 6.2 | 1 |
| 58 | f | - |  | 0 | 0 | 20 | 0 | 1 | 0 | 3.6 | 1 | 9.5 | 1 |
| 45 | f | - |  | 0 | 0 | 50 | 1 | 0 | 0 | 4.7 | 1 | 6.8 | 1 |
| 71 | f | - |  | 0 | 0 | 50 | 1 | 0 | 1 | 5.0 | 1 | 12.0 | 1 |
| 54 | f | - |  | 1 | 0 | 70 | 0 | 1 | 0 | 6.1 | 1 | 14.9 | 1 |
| 84 | f | - |  | 1 | 0 | 20 | 1 | 1 | 0 | 6.1 | 1 | 20.0 | 1 |
| 2 | f | - |  | 1 | 0 | 40 | 1 | 0 | 0 | 6.3 | 1 | 80.7 | 0 |
| 27 | f | - |  | 0 | 0 | 65 | 1 | 1 | 0 | 6.3 | 1 | 11.5 | 1 |
| 74 | f | - |  | 1 | 0 | 20 | 1 | 1 | 0 | 6.5 | 1 | 17.2 | 1 |
| 7 | f | - |  | 0 | 0 | 90 | 0 | 0 | 1 | 7.0 | 1 | 21.5 | 1 |
| 54 | f | - |  | 1 | 0 | 20 | 1 | 1 | 0 | 7.0 | 1 | 39.6 | 1 |
| 61 | f | - |  | 1 | 1 | 80 | 0 | 1 | 1 | 7.5 | 1 | 36.7 | 0 |
| 51 | f | - |  | 0 | 0 | 50 | 1 | 1 | 0 | 8.1 | 1 | 24.6 | 1 |
| 43 | f | - |  | 1 | 0 | 40 | 1 | 1 | 0 | 8.2 | 1 | 15.6 | 1 |
| 46 | f | - |  | 1 | 0 | 30 | 1 | 1 | 0 | 9.6 | 1 | 25.1 | 1 |
| 62 | f | - |  | 1 | 0 | 70 | 1 | 1 | 1 | 10.6 | 1 | 22.7 | 1 |
| 80 | f | - |  | 1 | 0 | 70 | 1 | 0 | 0 | 12.3 | 1 | 19.4 | 1 |
| 69 | f | - |  | 1 | 0 | 80 | 1 | 1 | 0 | 13.5 | 1 | 20.9 | 1 |
| 70 | f | - |  | 0 | 0 | 25 | 1 | 1 | 0 | 14.8 | 1 | 22.4 | 1 |

| | | | | | | | | | | | | | | |
|---|---|---|---|---|---|---|---|---|---|---|---|---|---|---|
| 56 | f | - | | 1 | 0 | 70 | 0 | 1 | 0 | 15.2 | 1 | | 20.2 | 1 |
| 14 | f | - | | 0 | 1 | 50 | 0 | 0 | 0 | 15.8 | 1 | | 24.6 | 1 |
| 33 | f | - | | 1 | 1 | 80 | 0 | 1 | 0 | 17.1 | 1 | | 23.6 | 1 |
| 79 | f | - | | 1 | 0 | 40 | 1 | 0 | 0 | 18.0 | 1 | | 19.2 | 1 |
| 55 | f | - | | 0 | 0 | 80 | 1 | 0 | 0 | 19.3 | 1 | | 22.0 | 0 |
| 55 | f | - | | 0 | 0 | 30 | 0 | 0 | 1 | 19.6 | 0 | | 19.6 | 0 |
| 60 | f | - | | 1 | 0 | 65 | 1 | 1 | 0 | 20.4 | 1 | | 29.2 | 1 |
| 63 | f | - | | 1 | 0 | 85 | 1 | 1 | 0 | 20.7 | 1 | | 26.1 | 1 |
| 83 | f | - | | 1 | 0 | 40 | 1 | 0 | 0 | 24.9 | 0 | | 26.2 | 1 |
| 35 | f | - | | 1 | 0 | 40 | 1 | 1 | 0 | 27.8 | 0 | | 36.1 | 0 |
| 41 | f | - | | 1 | 0 | 80 | 1 | 1 | 0 | 32.3 | 1 | | 36.2 | 0 |
| 70 | f | - | | 1 | 0 | 45 | 1 | 1 | 1 | 38.3 | 1 | | 48.8 | 1 |
| 40 | f | - | | 1 | 0 | 50 | 1 | 1 | 1 | 39.1 | 1 | | 53.0 | 0 |
| 42 | f | - | | 1 | 1 | 25 | 1 | 1 | 0 | 58.5 | 0 | | 66.9 | 0 |
| 49 | f | - | | 1 | 1 | 35 | 1 | 1 | 0 | 82.3 | 0 | | 82.3 | 0 |
| 85 | f | - | | 0 | 0 | 35 | 1 | 0 | 0 | 2.3 | 1 | | 3.0 | 1 |
| 20 | f | - | | 1 | 0 | 35 | 1 | 1 | 0 | 3.5 | 1 | | 18.0 | 1 |
| 58 | m | - | | 0 | 0 | 75 | 1 | 1 | 0 | 2.3 | 1 | | 14.6 | 1 |
| 67 | m | - | | 0 | 0 | 45 | 0 | 0 | 0 | 3.5 | 1 | | 5.4 | 1 |
| 73 | m | - | | 1 | 0 | 40 | 1 | 0 | 0 | 22.0 | 0 | | 22.0 | 0 |
| 57 | m | - | | 1 | 0 | 15 | 0 | 1 | 0 | 1.0 | 1 | | 3.9 | 1 |
| 48 | m | - | | 0 | 0 | 70 | 1 | 0 | 0 | 1.1 | 1 | | 8.7 | 1 |
| 55 | m | - | | 0 | 0 | 15 | 1 | 1 | 1 | 1.2 | 1 | | 12.1 | 1 |
| 65 | m | - | | 0 | 0 | 60 | 1 | 0 | 0 | 1.5 | 1 | | 6.5 | 1 |
| 41 | m | - | | 0 | 0 | 90 | 1 | 0 | 0 | 1.8 | 1 | | 3.6 | 1 |
| 11 | m | - | | 0 | 0 | 70 | 1 | 0 | 0 | 2.2 | 1 | | 2.2 | 1 |
| 65 | m | - | | 0 | 0 | 40 | 1 | 1 | 0 | 2.2 | 1 | | 4.1 | 1 |
| 33 | m | - | | 0 | 0 | 95 | 1 | 0 | 0 | 2.8 | 1 | | 3.3 | 1 |
| 38 | m | - | | 0 | 0 | 45 | 1 | 1 | 0 | 2.8 | 1 | | 22.8 | 1 |
| 70 | m | - | | 0 | 0 | 30 | 1 | 1 | 0 | 2.9 | 1 | | 12.2 | 1 |
| 62 | m | - | | 0 | 0 | 40 | 1 | 1 | 0 | 3.2 | 1 | | 14.8 | 1 |
| 56 | m | - | | 1 | 0 | 40 | 1 | 0 | 0 | 3.6 | 1 | | 6.7 | 1 |
| 64 | m | - | | 0 | 0 | 35 | 1 | 1 | 0 | 4.0 | 1 | | 12.8 | 1 |
| 78 | m | - | | 0 | 0 | 80 | 1 | 0 | 0 | 4.0 | 1 | | 24.6 | 1 |
| 60 | m | - | | 0 | 0 | 25 | 1 | 1 | 0 | 4.1 | 1 | | 15.9 | 1 |
| 58 | m | - | | 1 | 0 | 25 | 0 | 1 | 0 | 4.6 | 1 | | 23.1 | 1 |
| 61 | m | - | | 1 | 0 | 70 | 1 | 0 | 0 | 4.8 | 1 | | 10.5 | 1 |
| 69 | m | - | | 0 | 0 | 40 | 1 | 0 | 0 | 4.9 | 1 | | 8.5 | 1 |
| 54 | m | - | | 0 | 0 | 60 | 1 | 1 | 0 | 5.1 | 1 | | 10.6 | 1 |
| 74 | m | - | | 0 | 0 | 30 | 1 | 1 | 0 | 5.1 | 1 | | 16.5 | 1 |

| | | | | | | | | | | | | | |
|---|---|---|---|---|---|---|---|---|---|---|---|---|---|
| 56 | m | - | | 1 | 0 | 60 | 1 | 0 | 0 | 5.3 | 1 | 17.6 | 1 |
| 5 | m | - | | 0 | 0 | 15 | 1 | 0 | 1 | 5.4 | 1 | 20.1 | 1 |
| 61 | m | - | | 1 | 0 | 35 | 1 | 1 | 0 | 5.4 | 1 | 20.1 | 1 |
| 76 | m | - | | 0 | 0 | 20 | 1 | 1 | 0 | 5.4 | 1 | 6.3 | 1 |
| 38 | m | - | | 0 | 0 | 40 | 1 | 1 | 0 | 5.7 | 1 | 10.3 | 1 |
| 16 | m | - | | 0 | 0 | 50 | 0 | 1 | 0 | 5.9 | 1 | 5.9 | 1 |
| 26 | m | - | | 1 | 0 | 80 | 1 | 1 | 1 | 6.0 | 1 | 16.7 | 0 |
| 81 | m | - | | 1 | 0 | 30 | 1 | 0 | 0 | 6.4 | 0 | 6.4 | 0 |
| 68 | m | - | | 1 | 0 | 40 | 1 | 1 | 0 | 6.7 | 1 | 39.7 | 1 |
| 12 | m | - | | 1 | 0 | 35 | 0 | 0 | 0 | 7.2 | 1 | 14.0 | 1 |
| 60 | m | - | | 1 | 0 | 50 | 1 | 1 | 0 | 7.4 | 1 | 20.3 | 1 |
| 61 | m | - | | 0 | 0 | 30 | 1 | 0 | 0 | 7.5 | 1 | 9.9 | 0 |
| 56 | m | - | | 0 | 0 | 60 | 1 | 0 | 1 | 8.1 | 1 | 19.0 | 1 |
| 32 | m | - | | 1 | 1 | 70 | 1 | 0 | 1 | 8.2 | 1 | 10.7 | 1 |
| 9 | m | - | | 1 | 0 | 90 | 1 | 1 | 0 | 8.2 | 1 | 15.8 | 1 |
| 53 | m | - | | 1 | 0 | 80 | 1 | 1 | 0 | 8.7 | 1 | 17.2 | 1 |
| 30 | m | - | | 1 | 0 | 70 | 1 | 0 | 1 | 8.8 | 1 | 10.7 | 1 |
| 54 | m | - | | 0 | 0 | 25 | 1 | 0 | 0 | 9.6 | 1 | 15.2 | 1 |
| 77 | m | - | | 1 | 0 | 20 | 1 | 0 | 0 | 10.0 | 1 | 21.8 | 1 |
| 40 | m | - | | 1 | 0 | 25 | 0 | 0 | 1 | 10.3 | 1 | 20.8 | 0 |
| 47 | m | - | | 0 | 0 | 40 | 1 | 1 | 1 | 10.4 | 1 | 18.3 | 0 |
| 33 | m | - | | 1 | 0 | 40 | 1 | 1 | 0 | 11.2 | 1 | 21.5 | 1 |
| 71 | m | - | | 1 | 0 | 50 | 1 | 0 | 1 | 12.2 | 0 | 12.3 | 0 |
| 53 | m | - | | 0 | 0 | 30 | 1 | 0 | 0 | 12.8 | 1 | 15.3 | 1 |
| 13 | m | - | | 1 | 0 | 80 | 1 | 0 | 0 | 13.5 | 1 | 27.5 | 1 |
| 83 | m | - | | 1 | 0 | 50 | 1 | 0 | 0 | 13.9 | 1 | 13.9 | 1 |
| 54 | m | - | | 0 | 0 | 35 | 0 | 0 | 1 | 14.4 | 1 | 18.6 | 1 |
| 32 | m | - | | 0 | 1 | 45 | 0 | 0 | 0 | 14.8 | 1 | 17.6 | 1 |
| 57 | m | - | | 0 | 0 | 60 | 1 | 1 | 0 | 14.9 | 1 | 16.6 | 1 |
| 43 | m | - | | 0 | 0 | 20 | 1 | 0 | 0 | 15.9 | 1 | 23.7 | 1 |
| 53 | m | - | | 1 | 0 | 40 | 1 | 1 | 0 | 18.3 | 1 | 28.3 | 1 |
| 30 | m | - | | 0 | 1 | 60 | 1 | 1 | 0 | 21.8 | 1 | 23.1 | 1 |
| 44 | m | - | | 1 | 0 | 20 | 1 | 1 | 1 | 22.3 | 1 | 56.9 | 0 |
| 63 | m | - | | 1 | 0 | 70 | 1 | 1 | 1 | 22.6 | 1 | 25.2 | 1 |
| 60 | m | - | | 0 | 0 | 20 | 1 | 0 | 0 | 22.6 | 1 | 34.3 | 1 |
| 61 | m | - | | 1 | 0 | 20 | 1 | 1 | 0 | 23.3 | 1 | 40.3 | 1 |
| 36 | m | - | | 0 | 1 | 70 | 1 | 1 | 0 | 24.7 | 0 | 33.2 | 0 |
| 61 | m | - | | 1 | 0 | 45 | 1 | 0 | 0 | 26.1 | 1 | 34.0 | 1 |
| 72 | m | - | | 0 | 0 | 15 | 1 | 1 | 0 | 29.3 | 1 | 30.6 | 0 |
| 52 | m | - | | 1 | 0 | 50 | 1 | 1 | 0 | 68.5 | 0 | 75.3 | 1 |

KPS80, Karnofsky performance status ≥ 80; IDH1, isocitrate dehydrogenase 1; MSP, MGMT methylation specific PCR; PFS, Progression-free survival; OS, Overall survival
